# Recrystallization and Grain Growth in Cu-Cu Joints under Electromigration at Low Temperatures

**DOI:** 10.3390/ma16175822

**Published:** 2023-08-25

**Authors:** Shih-Chi Yang, Dinh-Phuc Tran, Chih Chen

**Affiliations:** Department of Materials Science and Engineering, National Yang Ming Chiao Tung University, Hsinchu 30010, Taiwan; scdic1201@gmail.com (S.-C.Y.); trandinhphuc1508@gmail.com (D.-P.T.)

**Keywords:** recrystallization and grain growth, electromigration, Cu-Cu direct bonding, elimination of bonding interface, advanced packaging

## Abstract

The behavior of recrystallization and grain growth was examined in Cu-Cu joints during electromigration at 150 °C. Recrystallization and grain growth were observed in all the joints after electromigration for 9000 h. Voiding was formed in Cu current-feeding lines and in bonding interfaces, and resistance increased with time due to the void formation. However, instead of rising abruptly, the resistance of certain Cu joints dropped after 7000 h. Microstructural analysis revealed that a large grain growth occurred in these joints at 150 °C, and the bonding interface was eliminated. Therefore, the electromigration lifetime can be prolonged for these joints.

## 1. Introduction

The three-dimensional integrated circuit (3D IC) has become a key technology for advanced packaging due to its ultra-high input and outputs (I/Os) density and enhanced reliability performance [1,2,3]. Traditionally, solder microbumps have been adopted for interconnects between chips. However, solder joints may fail due to bridging failure and side-wall wetting effects when the pitch of the joints is scaled down [4,5]. And the rapid formation of ductile intermetallic compounds (IMCs) will lead to the degradation of mechanical property and reliability performance [6,7]. To overcome the scale-limit issues, Cu-Cu bonding is emerging as a promising approach for interconnects, since it exhibits excellent electrical properties and is compatible with the processing temperature of state-of-the-art back-end-of-line (BEOL) technology [8,9,10]. Current 3D-stacked structures such as high bandwidth memory (HBM) are fabricated by using a novel Cu-Cu bonding scheme [11,12]. Furthermore, they have been implemented as the interconnections in back-illuminated CMOS image sensors (BI-CIS) and image signal processors (ISPs) [13,14,15].

To lower the thermal budget and bonding temperature, highly (111)-oriented nanotwinned Cu (NT-Cu) has been adopted in Cu-Cu direct bonding and the ultra-fine pitch damascene process [16,17]. Thanks to its high surface diffusivity and low oxidation rate [18,19], low-temperature Cu-Cu bonding could be achieved at 200 °C [20]. In consideration of the thermal behavior, NT-Cu is featured with an anisotropic grain growth behavior which can eliminate the interface of the Cu-Cu joints [21]. Furthermore, the recrystallization of Cu could eliminate the voids at the bonding interface, which leads to enhanced bonding quality [22]. In our previous study [23], single-crystal-like (SCL) Cu joints without bonding interfaces exhibited high resistance to thermal fatigue failure. Yet the temperature had to exceed 250 °C to grow the SCL Cu joints.

Electromigration (EM)-induced degradation is another critical issue in interconnects due to rapid scaling and the increased current stressing of the devices [24,25,26]. Common EM failures in solder interconnects includes void formation and under-bump-metallization (UBM) dissolution which may result in a rapid increase in resistance and finally cause open failure [27,28]. Regarding the continuous direct current through the joint, it was reported that recrystallization at the alloy and solder interface could be induced by current stressing incorporated with the generated Joule heat at 105.7 °C [29,30]. However, Cu has a higher melting point than solders. It was reported that Cu-Cu joints possess a much longer EM lifetime than solder joints [31,32]. Although several investigations have been conducted to study the EM of Cu-Cu joints [33,34], the influence of current-induced recrystallization at the Cu-Cu bonding interface on electrical properties has not been reported.

In this research, NT-Cu joints were successfully bonded at 200 °C. Other than accelerated EM conditions at high temperatures [35,36], EM tests were carried out at 150 °C which is closer to the user condition. Recrystallization was observed after current stressing at 150 °C for 7000 h. Interestingly, a drop in resistance was observed after prolonged current stressing. According to further microstructural analysis, the resistance reduction is due to recrystallization-driven grain growth and the elimination of the bonding interface.

## 2. Materials and Methods

**Figure 1:**
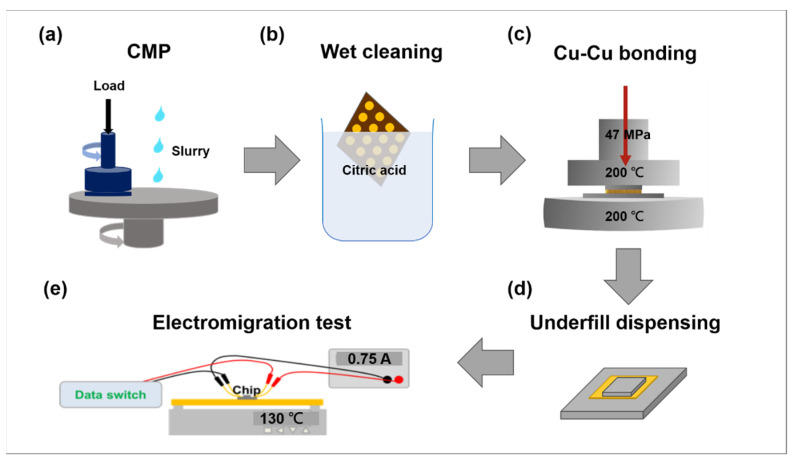
Process flow of this study. (**a**) The as-deposited 8-inch wafer underwent the CMP process. The surface roughness (*R*_q_) was around 5 nm. (**b**) Wet cleaning process. Both top dies and bottom dies were rinsed with citric acid for 30 s followed by a nitrogen purge. (**c**) Cu-Cu direct bonding. Dies were aligned and bonded with a die-to-die bonder under vacuum ambient. The bonding conditions were 200 °C and 1 h. The bonding pressure was 47 MPa. (**d**) The underfill-dispensing process was carried out before EM tests. (**e**) Electromigration tests’ setup. Samples were heated at 130 °C on the top of the hotplate. The applied current was 0.75 A generated by the power supply. Resistance was recorded by data switch.

**Figure 2:**
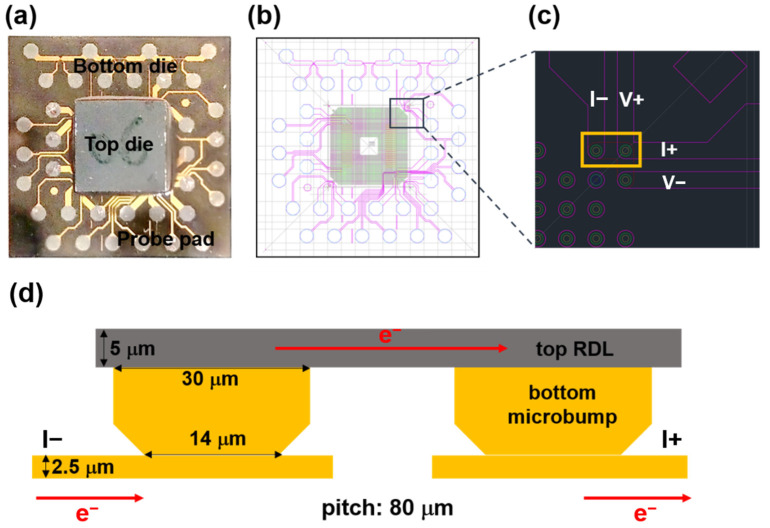
(**a**) Image of as-bonded samples after underfill dispensing. (**b**) Electrical layout designed for this study. Three pairs of Kelvin structures are included. (**c**) Enlarged schematic of Kelvin structure. A pair of Kelvin bumps which would undergo current stressing is labeled with a yellow rectangle. (**d**) Schematic of the current-stressed Kelvin structure. The electron flow is indicated by the red arrow.

The fabrication flow of NT-Cu joints is illustrated in Figure 1. Arrays of NT-Cu microbumps were firstly electroplated on 8-inch Si wafers. The electrolyte consisted of 50 g Cu, 100 g H_2_SO_4_, and 50 ppm of Cl^-^ ions. Then, the as-deposited wafers underwent chemical mechanical planarization (CMP) to obtain a flat surface with a roughness (*R*_q_) of 5 nm. Next, the 8-inch wafers were diced into small dies for subsequent cleaning and bonding processes. Each die was cleaned with citric acid for the removal of the oxide layer on the surface. Then, the dies were rinsed by deionized water and were purged by nitrogen before bonding. The Cu-Cu direct bonding process was performed by a die-to-die bonder (CA-2000VA, Bondtech Co., Ltd., Kyoto, Japan). Thermal compression bonding (TCB) was executed at 200 °C for 1 h at vacuum ambient. The bonding pressure was 47 MPa. Such a bonding condition was favorable for the good bonding strength and low thermal budget of the joints. According to our previous study [23], Cu-Cu joints could be bonded at 150 °C under a bonding pressure of 41.5 MPa. However, the Cu-Cu joints bonded at 200 °C and 41.5 MPa possessed a higher bonding strength and thermal fatigue resistance. Thus, a similar bonding condition (200 °C and 47 MPa) was selected for TCB in the current study. In order to prevent further oxidation issues during reliability tests and compensate for the mismatch of the coefficient of thermal expansion (CTE) between Cu and silicon, underfill (UF) was dispensed into the gaps between the joints. The bonded sample with UF protection is shown in Figure 2a.

Figure 2b exhibits the layout of the test vehicle used in this work. A Kelvin structure and daisy chain with 400 (DC400) and 40 (DC40) microbumps connected in series were designed for electrical measurements. A pair of Kelvin bumps were subjected to continuous current stressing during the EM test (Figure 2c). A schematic of the desired cross-section for failure analysis is illustrated in Figure 2d. The top die consisted of arrays of Cu redistribution lines (RDLs). The width and thickness were 45 µm and 5 µm, respectively. The bottom die consisted of both Cu microbumps and Cu traces. The microbumps were 30 µm in diameter and 10 µm in height. The pitch was 80 µm. A passivation (PSV) opening surrounded by poly-benzoxazole (PBO) was formed during the lithography steps. The opening was 14 µm in diameter, which connected with a 2.5-µm thick Cu trace. For the Kelvin structure, Cu traces were 125 µm in length and were connected to the probe pads for resistance measurements.

To evaluate the bonding quality of the bonded joints, a four-point probe technique was carried out to measure the initial resistance of the as-bonded samples. The applied current varied from −0.5 A to 0.5 A with 1000 data points recorded for I–V characterization. The electrical resistance of the as-bonded samples ranged from 2 mΩ to 3 mΩ. The Cu joints were classified based on the differences in their initial values. The Type I Cu joints possessed a higher initial resistance (2.8 mΩ on average), whereas the Type II Cu joints showed a lower initial resistance (2.3 mΩ on average). To execute the EM tests, the bonded samples were placed on a hotplate and were connected to a power supply for constant-current stressing. The temperature of the hotplate was controlled at 130 °C. A constant current of 0.75 A was used during EM tests. The calculated current density at the bonding interface was 105 A/cm^2^. The maximum current density was located at the Cu trace of the bottom die. It was 1.1 × 10^6^ A/cm^2^, which is about 10 times larger than the current density at the bonding interface. In consideration of the Joule heating effect, a temperature coefficient of resistance (TCR) calibration was carried out before EM tests. The actual temperature of the chip was measured as 150 °C, which means that the temperature increase due to Joule heating was close to 20 °C. The resistance of the Cu joints was recorded every 30 s during the EM tests.

In this investigation, prolonged EM tests of about one year of current stressing were performed, because the test temperature was as low as 150 °C. Samples were grinded and polished to the desired location of the Kelvin structure for the failure analysis. A focused ion beam (FIB) was employed to observe the microstructure of the Cu joints. Afterwards, electron backscattered diffraction (EBSD) analysis was conducted using a scanning electron microscope to detect the crystallographic grain distributions and to characterize the microstructure at the bonding interface.

## 3. Results and Discussion

**Figure 3:**
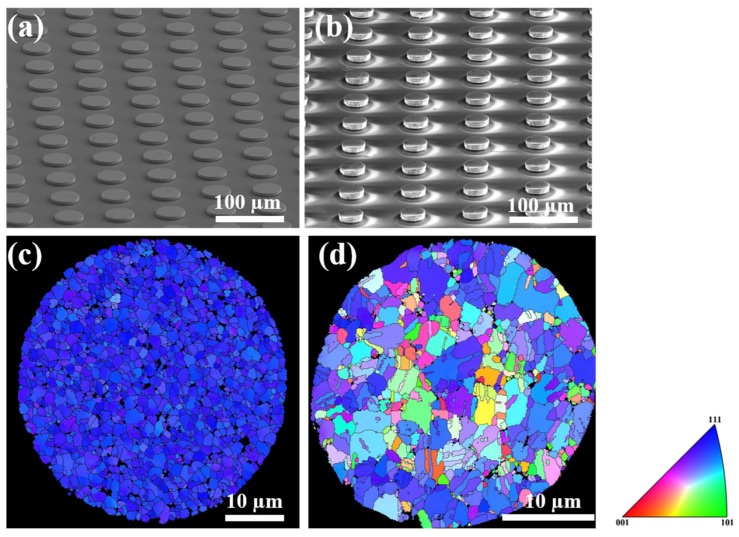
(**a**) SEM image of top-die RDLs after CMP. The diameter is 45 µm. (**b**) SEM image of bottom-die microbumps after CMP. The diameter is 30 µm. (**c**) EBSD image of top-die RDLs. The ratio of the <111>-preferred orientation is 99.6%, calculated by OIM. (**d**) EBSD image of bottom-die microbumps. The ratio of the <111>-preferred orientation is 43.8%, calculated by OIM.

Arrays of NT-Cu RDLs and microbumps after CMP are exhibited by second electron images in Figure 3a,b, respectively. The crystallographic orientation of the Cu surface was detected from the normal direction by EBSD analysis. As shown in Figure 3c, the top die RDLs possess as high as 99.6% of (111)-oriented grains at the surface. As aforementioned, such a highly (111)-preferred orientation could be beneficial to low-temperature Cu-Cu bonding since the <111> plane owns the highest surface diffusivity in face-centered cubic (FCC) materials [16,18]. On the other hand, the (111)-oriented percentage of the bottom die microbumps is only 43.8%, as shown in Figure 3d. The decreased fraction of (111)-oriented grains is due to the geometrical effect of the arc of the PSV. Nevertheless, we could still achieve low-temperature Cu-Cu bonding at 200 °C.

**Figure 4:**
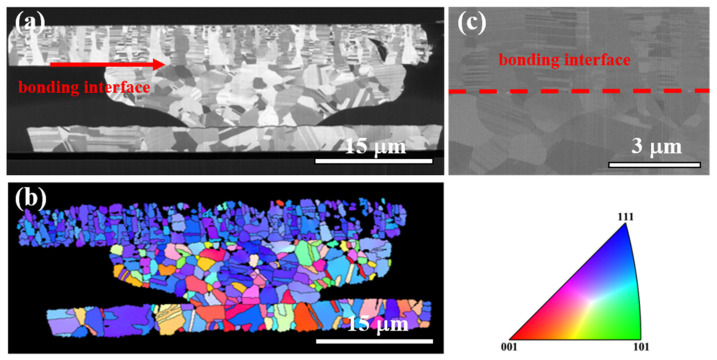
Microstructure analysis of the as-fabricated Cu joint. (**a**) Cross-section FIB ion image. The bonding interface is pointed out by the red arrow. (**b**) Corresponding cross-sectional EBSD image for the Cu joint in Figure 1a. Inverse pole figure is shown. (**c**) FIB electron image at the bonding interface of the Cu joint in Figure 1a. The bonding interface is indicated by the red dotted line.

**Figure 5:**
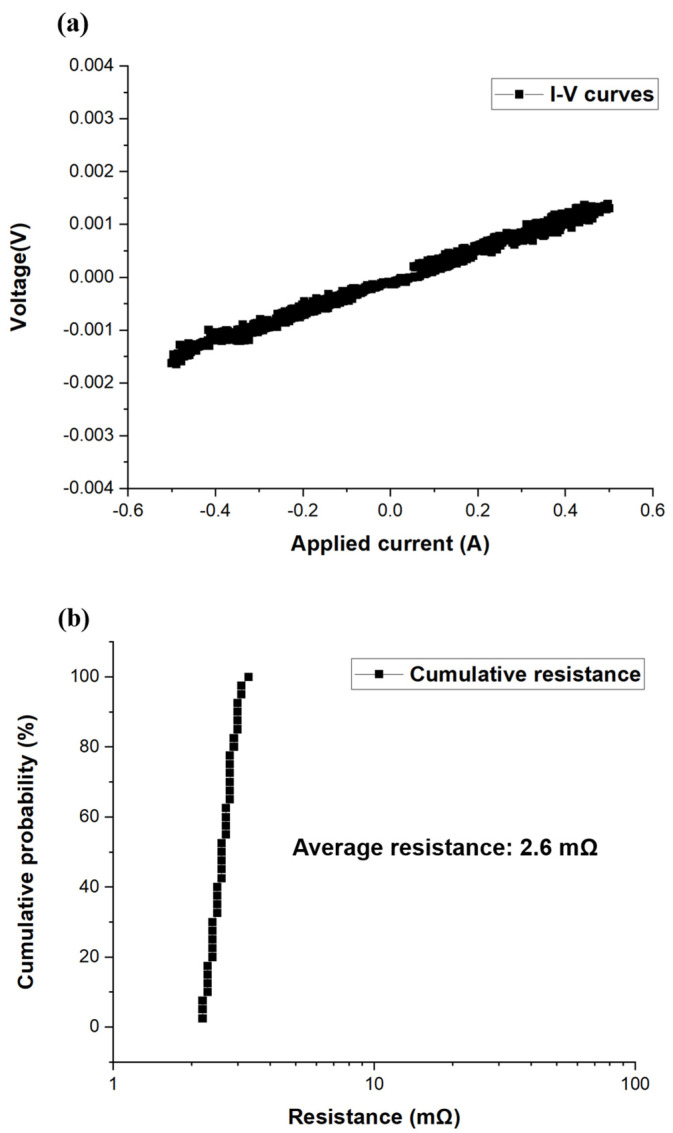
Electrical analysis of the as-fabricated Cu joint. (**a**) Current-voltage (I–V) characterization of a single Kelvin bump. (**b**) Cumulative probability of resistance.

In this investigation, the Cu joints could be bonded at 200 °C for 1 h, and no obvious grain growth was observed. The microstructure of the as-bonded NT-Cu joints is presented in Figure 4a. The bonding interface of the Cu joint is labeled by a red arrow. Columnar nanotwinned grains can be observed in the top die, and these are highly (111)-oriented grains as revealed by the analysis of cross-sectional EBSD (Figure 4b). An enlarged FIB electron image of the bonding interface is exhibited in Figure 4c, and the bonding interface is indicated by a red dotted line. There is no obvious recrystallization or grain growth occurring at the bonding interface, since the bonding process is only 1 h. The average resistance of the as-bonded Kelvin bumps is 2.6 mΩ. And the cumulative resistance plots in Figure 5a exhibit a small variation among all 40 of the Kelvin bumps. The calculated specific contact resistance is only 1.84 × 10^−8^ Ω·cm^2^, which indicates a good bonding quality. Furthermore, the current-voltage (I–V) characterization of the single Kelvin bumps (Figure 5b) also shows a great electrical performance. Therefore, one could expect that the promising bonding quality of the as-bonded joints is strong enough against continuous current stressing under EM.

**Figure 6:**
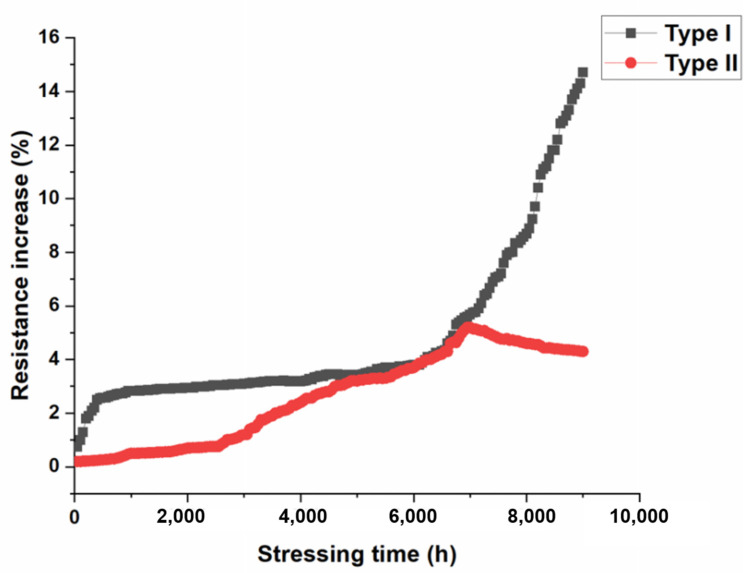
Plot of the resistance as a function of EM stressing time. Vertical axis represents the percentage of resistance increment (∆*R*/*R*_0_); Two distinguishable EM behaviors were observed.

It is intriguing that the resistance of the Cu joints dropped after current stressing at 150 °C for 7000 h. Figure 6 depicts the curves of the resistance increment (∆*R*) profile with the current stressing time (*t*). Two types of resistance curves were observed after 9000 h of EM tests at 150 °C. Such a typical difference in the EM behaviors of these two types could be attributed to the initial bonding quality of the as-bonded joints. We found that the bonding quality of the Type II joints was better than that of Type I. Thus, the EM performances of the Cu-Cu joints could be correlated to their initial bonding quality. The Type I ∆*R*-*t* curve shows a gradual rise from the initial stage of the EM test, and then an abrupt increase in resistance after 6000 h. Finally, the resistance increment reached 15% of its initial value, which is a typical failure criterion for microelectronic interconnects. This is the typical EM behavior for interconnects [37]. Further investigation into the reason for the increased resistance will be discussed in the next section of microstructural analysis. However, we observed an interesting EM behavior, which has not been reported before. As illustrated by the Type II curve in Figure 6, the resistance rose gradually from the initial stage to about 7000 h, and the resistance increment was approximately 5% at 7000 h. Surprisingly, instead of a rapid rise in resistance, a slight reduction in resistance was observed. The resistance increment dropped to 4% when the current-stressing time reached 9000 h.

**Figure 7:**
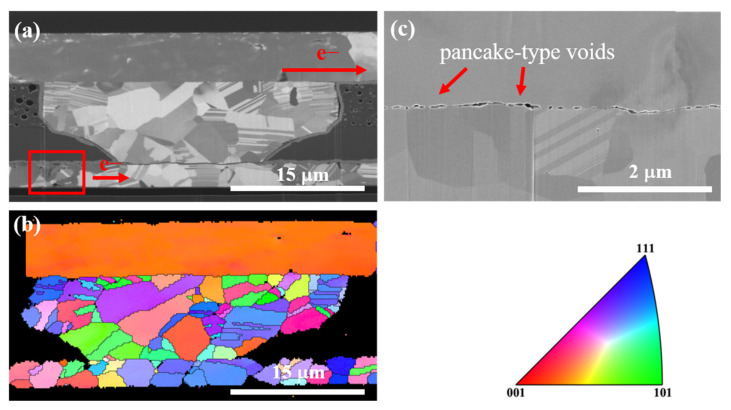
Microstructure analysis for the Type I Cu joint after the EM test at 150 °C for 9000 h. (**a**) Cross-section FIB ion image. (**b**) Corresponding EBSD image for the Cu joints in Figure 3a. Inverse pole figure is shown. (**c**) Enlarged FIB electron image at the bonding interface for the Cu joint in Figure 3a.

Figure 7 presents the microstructural analysis of the Cu joints corresponding to the Type I sample in Figure 6. Figure 7a is the FIB ion image of the Kelvin bump undergoing current stressing at 150 °C for 9000 h. The electron flow was upward as indicated in the figure. As pointed out by the red rectangle, several voids formed due to the electron wind force at the Cu trace near the entrance of the PSV opening at the cathode side. This is reasonable, since the maximum current density was located at the Cu trace of the bottom die. Furthermore, pancake-type void formation was observed at the bonding interface, as shown in Figure 7c. Electron wind force and thermal-induced tensile stress may be the main reasons for the void formation at the bonding interface [36]. Figure 7b is the corresponding EBSD image, which indicates that recrystallization and grain growth took place in the Cu RDL on the top die. Yet the grain growth stopped at the bonding interface. It can be concluded that the resistance increase is attributed to the void formation at the Cu RDL and at the bonding interface.

**Figure 8:**
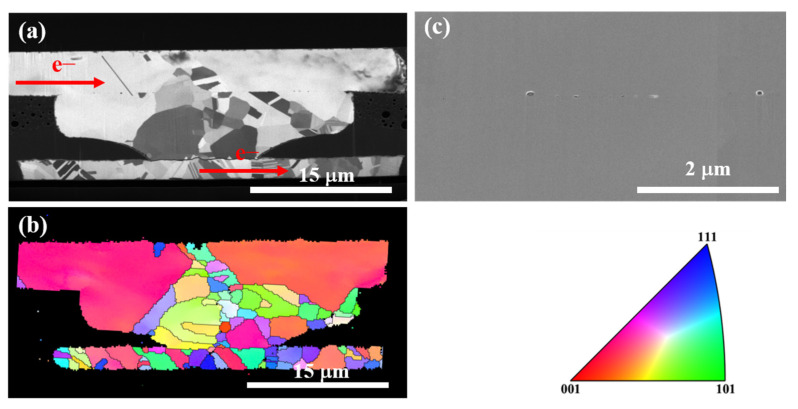
Microstructure analysis of the Type II Cu joint underwent the EM test at 150 °C for 9000 h. (**a**) Cross-section FIB ion image. (**b**) Corresponding cross-sectional EBSD image for the Cu joint in Figure 4a. Inverse pole figure is shown. (**c**) Enlarged FIB electron image at the left side of the Cu joint in Figure 4a.

Interestingly, recrystallization and grain growth occurred in the Type II Cu joints, and grain growth extended across the bonding interface. Figure 8 shows the microstructural analysis of the Cu joints corresponding to the Type II joints in Figure 6. Figure 8a presents the FIB ion image of the Kelvin bump with a downward electron flow. No obvious cracks were observed in the Type II joints, which were comparable to those of Type I. Several spherical voids were found in the joints, as shown in Figure 8c. These voids originally existed at the bonding interface after TCB. Once the bonding interfaces were eliminated by recrystallization and grain growth during EM tests, they might have been trapped inside the Cu grains. Furthermore, the bonding interface was eliminated at the periphery of the joint where the current density was higher (Figure 8c) [38]. The elimination of the bonding interface triggered by recrystallization and large grain growth of NT-Cu was further confirmed by the EBSD analysis in Figure 4c. Such a phenomenon can be explained by the combination of EM and Joule heat-induced recrystallization revealed by the previous study [29]. It has been reported that a large strain can be generated in metals by electric current stressing [39]. This results in a high dislocation density which leads to recrystallization as demonstrated in the previous study [40]. Moreover, a similar phenomenon was reported in another study showing that the resistance decrease could be ascribed to the improved Cu rearrangement at the interface [32]. As a result, since the bonding interface vanished, the measured resistance of the Cu joints reduced. This finding is crucial for Cu joints, because their EM lifetime can be prolonged when the bonding interface is eliminated.

**Figure 9:**
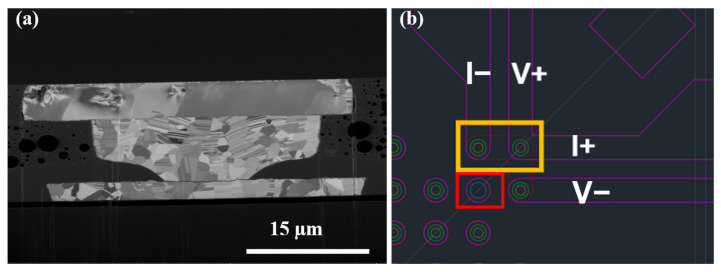
(**a**) Typical FIB image of the dummy Cu joints which underwent a similar annealing history without EM stressing. (**b**) The dummy Cu joint located near the Kelvin structure is denoted by the red rectangle.

Ong et al. reported that large grain growth can be triggered in the Cu joints at a temperature above 250 °C for 1 h [23]. In the present study, the temperature was lowered to 150 °C due to EM. As a controlled sample, we also examined the microstructure of the Cu joints that underwent a similar annealing history but without EM. The typical FIB image of the dummy Cu joints is shown in Figure 9. The dummy Cu joint without electric current (Figure 9b) is denoted by the red rectangle located near the Kelvin structure (the yellow rectangle). Only recrystallization and grain growth were observed in the Cu bumps in the top die. Therefore, the wind force of EM may play a critical role in the grain growth across the bonding interface.

It is noteworthy that the Cu joints of Type II had a lower initial resistance value than that of Type I joints. The average initial resistance for Type II Cu joints was 2.3 mΩ, whereas it was 2.8 mΩ for the Type I Cu joints. The bonding quality of Type II joints may be better than that of Type I, which means the bonding interface in Type II joints may have fewer Cu oxides. Therefore, grain growth may extend across the bonding interfaces and the resistance may be reduced. Nevertheless, a systematic study needs to be performed to correlate the initial interfacial microstructures on grain growth during EM.

## 4. Conclusions

To summarize this study, NT-Cu joints could be well-bonded at 200 °C for 1 h. The average contact resistance of the as-bonded joints was as low as 2.6 mΩ. Most importantly, recrystallization-induced resistance reduction in the Cu joints during current stressing is demonstrated in this study. The elimination of the bonding interface of the Cu joints was observed after EM at 150 °C for 9000 h, which was triggered by the recrystallization and large grain growth of the NT-Cu joints. On the other hand, the resistance of the Cu joints without large grain growth across the bonding interface continued to increase with time, leading to the failure of the joints.

## Data Availability

Not applicable.
